# The Anisotropic Distortional Yield Surface Constitutive Model Based on the Chaboche Cyclic Plastic Model

**DOI:** 10.3390/ma12030543

**Published:** 2019-02-12

**Authors:** Jianyun Chen, Keshi Zhang, Zheng Kuang, Guijuan Hu, Qiao Song, Yanjun Chang

**Affiliations:** 1 College of Civil Engineering and Architecture, Key Laboratory of Disaster Prevention and Structural Safety of Ministry of Education, Guangxi Key Laboratory of Disaster Prevention and Engineering Safety, Guangxi University, Nanning 530004, China; jianyun_chen@126.com (J.C.); zhangks@gxu.edu.cn (K.Z.); kuangzheng256@163.com (Z.K.); guxier@163.com (Q.S.); 2 School of Landscape Architecture, Zhejiang A & F University, Hangzhou 311300, China; hgj1973@sina.com

**Keywords:** anisotropic yield, constitutive model, subsequent yield surface, distortional yield surface

## Abstract

Considering the cross effect in the evolution of subsequent yield surfaces for metals, an anisotropic distortional yield surface constitutive model is developed. By introducing an anisotropic distortional hardening function into the isotropic hardening part of the classical Chaboche rate-dependent constitutive model, the plastic-deformation-induced distortional and anisotropic hardening behaviors of subsequent yield surfaces are characterized. The experimental data of distortional yield surfaces for T2 pure copper under three different loading paths, including pre-tension, pre-torsion, and pre-tension-torsion proportional loading of 45-degree, are simulated by implementing the models into a numerical user defined material (UMAT) procedure based on the ABAQUS finite element package. To validate the anisotropic plastic model, the simulated yield surfaces are compared with experimental observations and predicted results for a crystal plasticity model and good agreement are noted. The simulations demonstrate that the proposed model can accurately capture the characteristics of the distortional yield surface and the anisotropic hardening process of the yield surface. Moreover, the distortional shapes of experimental subsequent yield surfaces in loading direction and opposite direction can be better revealed by the anisotropic plastic constitutive model than the crystal plastic constitutive model.

## 1. Introduction

It has been long observed that plastic deformations induce anisotropy in initially isotropic materials [1,2], which has been a challenge for the traditional plastic constitutive theory to describe. The study of the evolution law of yield surface relies on the establishment of a plastic constitutive model, which serves as a basis for the analysis of macroscopic test data and verification of the applicability and validity of the model by multiaxial tests. As loading applied to the material increases, further deformation takes place, the stress state reaches the subsequent yield point, and there exists a subsequent yield surface for three-dimensional loading in the stress space. The evolution forms of a subsequent yield surface are decomposed into expansion and movement of the yield surface in classical plasticity theory, which correspond to isotropic hardening and kinematic hardening, respectively. Isotropic hardening and kinematic hardening can be characterized by a yield surface radius and a back stress in the von Mises theory. Until now, researchers have conducted many studies on plastic behaviors of metals [3,4,5,6,7].

**Figure 1:**
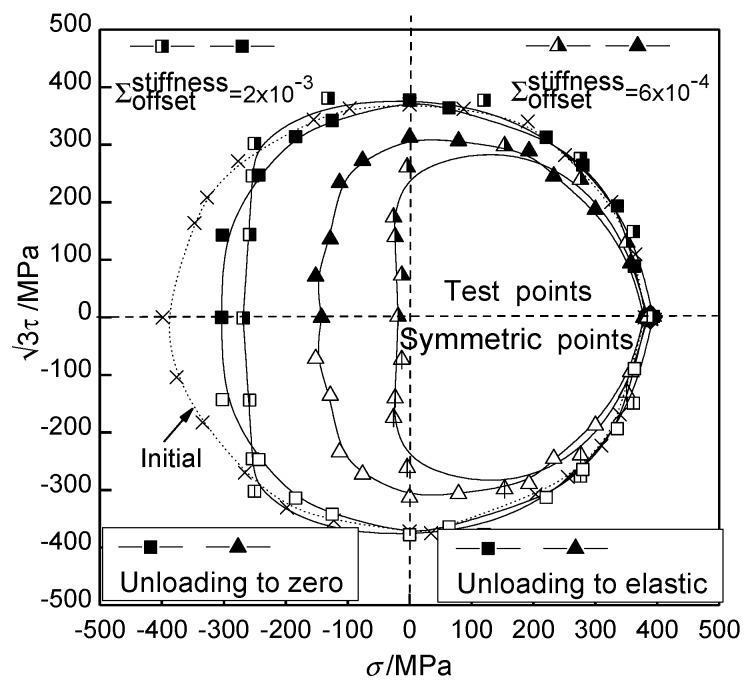
Schematics of subsequent yield surface of 45 steel on the stress plane under unloading to elastic range after preloading along tension direction [17].

**Figure 2:**
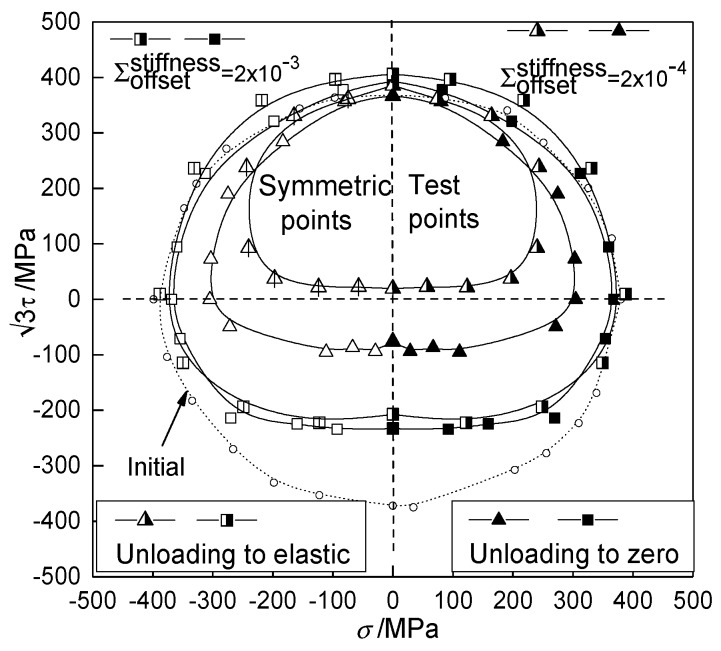
Schematics of subsequent yield surface of 45 steel on the stress plane under unloading to elastic range after preloading along torsion direction [17].

Chaboche [8,9,10] proposed a more general cyclic plasticity constitutive model on the basis of Armstrong and Frederick (A-F model) [11], which can better reflect the cyclic hardening and cyclic softening behaviors of materials under proportional loading. The experiments on various metals from Phillips [12], Wu [13], and Khan [14] showed distortion of the yield surface characterized by a region of high curvature along the direction of loading and a region of flattening on the opposite side. Moreover, the distortion of the subsequent yield surface varies for different materials. Hu Guijuan and Zhang Keshi [15,16,17] systematically studied the evolution behaviors of the yield surfaces for copper, steel, and aluminum under tension-shear loading. It was shown that the shape of the measured yield surface is related to the offset strain of the yield point. The subsequent yield surface defined by a small offset strain develops an obvious “sharp point”. The shape of the yield surface is close to the cylindrical surface with increases of offset strain, which is consistent with the circle yield surface described by Mises yield criterion. Typical distortional subsequent yield surfaces under pretension and pretorsion loadings are shown in Figure 1 and Figure 2 [17] for 45 steel.

Since the initial and subsequent yield surfaces described by the von Mises theory are cylindrical surfaces in principal stress space, the traditional models cannot describe the cross effect (the sharp point in pre-deformation direction and the relatively flat bottom at its opposite direction) of a yield surface accurately. Many researchers have studied the anisotropic evolution of a yield surface by the macroscopic method [18,19,20,21] and the mesoscopic method [22,23], respectively. In classical plasticity theory, the anisotropy is reflected by the translation and distortion of the yield surface. Francois [24] modified the classical von Mises yield criterion by replacing the usual stress deviator with distortional stress tensor $S_{d}$ and described the yield surface as an “egg shape” to reduce the difference between simulation and experiment. Shi Baodong et al. [25] improved the theoretical model by supplementing the distortion factor with the classical plastic constitutive theory. Fu Qiang et al. [26] established a slip element model to simulate the subsequent yield surface evolution based on the mechanism of slip deformation. Chen Yuan et al. [27] proposed the mathematical models for various deformation mechanisms of slip, twinning, and detwinning to capture the Bauschinger effect under cyclic loading. Wen et al. [28] used the self-consistent model to determine the yield characteristics of polycrystalline materials under different loading conditions. The crystal plasticity method is powerful enough to simulate the inhomogeneous deformation and anisotropy induced by pre-deformation [16,29,30,31]. The crystal plasticity constitutive model follows continuum mechanics and considers the slip mechanisms at grain-level by introducing resolved shear stresses along different slip orientations and the nonlinear hardening of back stress. Zhang Keshi et al. [16,17] adopted the Voronoi polycrystalline representative volume element to compute the subsequent yield surfaces of pure copper and aluminum under different proportional pre-loadings and tension-compression cyclic loading. The results showed that the anisotropic distortion of the yield surface can be characterized with the polycrystalline crystal plastic model. Due to the higher computational cost of polycrystalline analysis of subsequent yield behavior of metals, it is difficult to apply this approach directly in engineering structural analysis. A constitutive model presenting the anisotropic evolution of yield surface under the hypotheses of continuous and uniform deformation will be more accurate and efficient in engineering application.

The anisotropic distortion of the subsequent yield surface has an obvious difference in metals, including different work hardening aluminum alloy [18,32]. Taking the subsequent yield of T2 pure copper behavior into account, a macroscopic constitutive model reflecting the distortional yield surface induced by plastic deformation is put forward under the hypothesis of continuous uniform in material. The main research work includes the following: (1) a distortional hardening multiplier in conjunction with an anisotropic degradation factor are introduced into the isotropic hardening term of the yield function, and an anisotropic distortional yield surface model considering cross effect is proposed; (2) the yield surfaces simulated by the anisotropic plastic constitutive model are compared with the experimental results for T2 pure copper under pre-deformation, the simulations of the Chaboche cyclic plastic model and the crystal plasticity model. The model parameters are calibrated and the reasonability of the anisotropic model is verified.

## 2. Theoretical Model

The strain tensor can be decomposed into elastic $\varepsilon^{e}$ and plastic $\varepsilon^{p}$ parts under a small strain assumption as:

(1) $$\varepsilon =\varepsilon^{e}+\varepsilon^{p}.$$

According to Hooke’s law, the relationship of stress and strain is expressed as:

(2) $$\sigma =D:\varepsilon^{e},$$

where the fourth-order tensor D represents the material stiffness. The plastic constitutive model concentrates on the calculation of plastic deformation, and the incremental theory considers the influence of loading history on present deformation. The plastic strain rate ${\dot{\varepsilon }}^{p}$ of classical plastic theory in regards to rate-independent model is given by:

(3) $${\dot{\varepsilon }}^{p}=\sqrt{\frac{3}{2}}\dot{p}n=\frac{3}{2}\dot{p}\frac{\partial F_{y}}{\partial \sigma },$$

where $\dot{p}$ is the accumulated equivalent plastic strain rate, $F_{y}$ is the loading potential function, and a second-order tensor n is the plastic flow direction, which is calculated by the partial differential of the loading potential function according to Equation (22).

For a rate-dependent model, the equivalent plastic strain rate $\dot{p}$ and plastic strain rate ${\dot{\varepsilon }}^{p}$ can be expressed as:

(4) $$\dot{p}={\langle \frac{F_{y}}{K}\rangle }^{m},$$

(5) $${\dot{\varepsilon }}^{p}=\sqrt{\frac{3}{2}}\dot{p}n=\frac{3}{2}{\langle \frac{F_{y}}{K}\rangle }^{m}\cdot \frac{\partial F_{y}}{\partial \sigma },$$

where the symbol < > is the MacCauley bracket, K,n are the parameters reflecting the material viscosity. $F_{y}$ is the loading potential function, which is same as the yield function on the premise of the associative flow. The second-order tensor above n is the plastic flow direction, as noted above.

The yield function is the foundation in plastic hardening and flow analysis, thus a proper yield function should be developed and chosen to characterize most features of the subsequent yield surface due to the diversity yield behavior of the numerous materials. Based on the classical von Mises criterion, the yield function of mixed hardening mode is defined by:

(6) $$f=\left\|s-\alpha \right\|-R-\sigma_{y}=0,$$

where s, α are the deviatoric stress and back stress, respectively, $\sigma_{y}$ represents the yield stress corresponding to zero plastic strain, and R is the isotropic hardening function. For mixed hardening, the subsequent yield surfaces evolve as the expansion (i.e., isotropic hardening) and translation (i.e., kinematic hardening due to the evolution of back-stress) constitutive model.

The most common kinematic rule is the linear one due to Prager [3], given as:

(7) $$d\alpha =\frac{2}{3}Cd\varepsilon^{p}.$$

A practical way to describe a nonlinear kinematic hardening—giving rise to a correct modelization of cyclic loops—consists of introducing an evanescent memory of the plastic strain path, as initially proposed by Armstrong and Frederick [4]. This nonlinear kinematic effect appears in the internal stress equation:

(8) $$d\alpha =C\left(\frac{2}{3}ad\varepsilon^{p}-\alpha dp\right),$$

where the first term corresponds to the Prager’s linear rule and the second term to the evanescent strain memory, which will gradually weaken and disappear as the cumulative plastic strain increases; the cumulated plastic length is defined, for example, by:

(9) $$dp=\sqrt{\frac{2}{3}d\varepsilon^{p}:d\varepsilon^{p}}.$$

Chaboche et al. [8] proposed a very good continuous description of cyclic loading of materials, obeying a combination of isotropic and nonlinear kinematic rules by introducing many strain memory items. The back stress is decomposed into several individual variables, and the general form is:

(10) $$\alpha =\sum_{i=1}^{M}\alpha_{i},\;{\dot{\alpha }}_{i}=C_{i}\left(\frac{2}{3}a_{i}{\dot{\varepsilon }}^{p}-\alpha_{i}\dot{p}\right),$$

where M denotes the number of back stress components. Considering the description ability and conciseness of the model, M=2 is adopted in this paper. $a_{i}$ and $C_{i}$ are the material constants responsible for the evolution of the back stress.

The isotropic hardening function R of Equation (6) is given as:

(11) R=Q[1−exp(−b⋅p)],

where Q is used to describe the saturation value of an isotropic hardening function, b is a parameter describing the relationship between yield surface radius and plastic strain, and p is the accumulated equivalent plastic strain.

Subjected to the initial conditions:

(12) $$\alpha_{i}\left(0\right)=0,\;p\left(0\right)=0,\;R\left(0\right)=0.$$

Vincent [19] established a general cyclic plastic model by introducing 25 distortional variables to replace the classical back stress components. Considering the rotation of principal stress axes in the loading direction, this model is suitable for exhibiting the ratcheting behavior under multi-axial loading. Based on the contribution of Feigenbaum [20], the distortional hardening model of metal was proposed by implementing the fourth-order anisotropic evolution tensor into the classical Hill orthogonal anisotropic yield criterion in thermodynamic framework. The yield function is defined as:

(13) $$f=\left(s-\alpha \right):\left[\frac{3}{2}\bar{I}+\left(\frac{s-\alpha }{\left\|s-\alpha \right\|}:\alpha \right)A\right]:\left(s-\alpha \right)-k^{2}=0,$$

where s, α are deviatoric stress and back stress of yield function, respectively, $\bar{I}$ is the fourth-order unit stress deviatoric tensor, and A is the fourth-order anisotropic distortional hardening tensor.

Francois [24] replaced the deviatoric stress tensor with the distortional stress tensor in the yield function of the classical mixed hardening mode, and the distortional yield function can be defined as:

(14) $$f=\left\|s_{d}\left(s,\alpha ,R\right)-\alpha \right\|-R-\sigma_{y}=0,$$

where s, α are deviatoric stress and back stress of yield function, respectively, $\sigma_{y}$ represents the yield stress corresponding to zero plastic strain, and R is the isotropic hardening function. $s_{d}$ is the distortion stress tensor calculated by deviatoric stress s, back stress α, and isotropic hardening function R.

Introducing the anisotropic distortional function H into the yield function of classical mixed hardening model by combining the isotropic hardening and the kinematic hardening, which characterize, respectively, the expansion and translation of subsequent yield surface, the anisotropic yield function can be expressed as:

(15) $$F_{y}=\left\|s-\alpha \right\|-\left(\sigma_{0}+R\right)H\left(w,d\right),$$

where s, α are deviatoric stress and back stress of yield function. $\sigma_{0}$ represents the yield stress corresponding to zero plastic strain. H is the anisotropic distortional function determined by anisotropic distortional factor d and the anisotropic degradation factor w. In this presentation, the nonlinear kinematic hardening rate of yield surface, which determines the evolution of back stress (center of yield surface), is described as Equation (10), and the isotropic hardening of yield surface R is given by Equation (11). The values for the corresponding coefficients $a_{i}$ and $C_{i}$ are indicated in Table 1.

**Figure 3:**
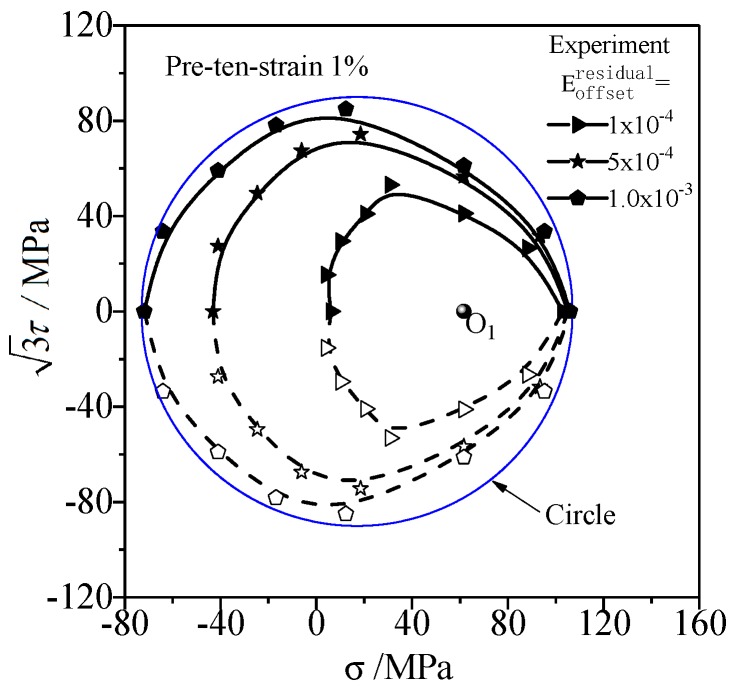
The experimental subsequent yield surfaces of T2 copper on the $\sigma -\sqrt{3}\tau$ stress plane with 1% pretension equivalent strain and unloading to 61.8 MPa [16].

The anisotropic distortional function H in Equation (15) can be summarized as:

(16) $$H\left(d,w\right)=\left\{ \begin{array}{ll} 1, & Preloading\;and\;unloading\\ d+\left(1-d\right)\frac{\left\|\alpha -\alpha_{unloading}\right\|}{w\cdot \left\|\alpha_{unloading}\right\|}, & Reloading\;and\;\frac{\left\|\alpha -\alpha_{unloading}\right\|}{w\cdot \left\|\alpha_{unloading}\right\|}\leq 1\\ 1, & Reloading\;and\;\frac{\left\|\alpha -\alpha_{unloading}\right\|}{w\cdot \left\|\alpha_{unloading}\right\|}\geq 1 \end{array}\right.,$$

where α is the back stress during reloading, and it is constant when the stress state is within the yield surface. The material yields with the increase of reloading load and back stress α evolves with the following Equation (10). The fourth-order tensor $\alpha_{unloading}$ is the back stress corresponding to the unloading stress followed by preloading in subsequent yield surface test, which is related to preloading path and preloading load. $\alpha_{unloading}$ remains the constant during elastic deformation.

(17) $$\alpha_{unloading}=\left\{ \begin{array}{ll} 0, & Preloading\\ \alpha_{unloading}, & Unloading\\ \alpha_{unloading}, & Reloading\;without\;unloading\\ \alpha_{new\;unloading}, & New\;unloading \end{array}\right.,$$

where w is the anisotropic degradation factor, which describes the evolution from anisotropy to quasi-isotropy with regard to yield function. It can be calibrated by the evolution test data of yield surface under different offset strains. If the anisotropic degradation factor w is very big, the subsequent yield surface presents only the proportional expansion of the initial anisotropic yield surface, i.e., the anisotropy of yield keeps stable. On the other hand, the initial anisotropic yield surface evolves toward the isotropic yield surface when the anisotropic degradation factor w is a smaller value, i.e., the anisotropy of yield degrades. An appropriate anisotropic degradation factor w is attempted based on the comparison of the evolution progress between the subsequent yield surface simulated and the experimental results for different given offset strains (confer the data in Figure 3). The anisotropic distortional function H varies from d to 1 with the increase of reloading load. When the back stress reaches a certain critical value, which means $\left\|\alpha -\alpha_{unloading}\right\|=w\cdot \left\|\alpha_{unloading}\right\|$ and H=1, the anisotropic distortional yield surface evolves into a yield surface without distortion.

The shape of subsequent yield surface under reloading tends to be circular in $\sigma -\sqrt{3}\tau$ stress space with the increase of the offset strain [15,17]. The subsequent yield surfaces of T2 pure copper under different offset strains [16] are shown in Figure 3. The results show that the anisotropy of the subsequent yield surfaces degrades gradually, and the subsequent yield surfaces approach a circle.

Noting the cross effect and anisotropic characteristics of metal subsequent yield surface test in Figure 3, the anisotropic distortional factor d in anisotropic distortional function H can be given as:

(18) $$d=\left[1-\beta_{1}+\beta_{1}{\left(\cos\theta \right)}^{3}\right]\left[1+\beta_{2}{\left(\sin\theta \right)}^{2}\right],$$

where $\beta_{1},\beta_{2}$ are the parameters of the cross effect, which can be calibrated by the characteristic of distortional subsequent yield surface corresponding to the small offset strain under tension and torsion loading (cf. Section 3.4). Anisotropic distortional yield surface degenerates into the circular mixed hardening yield surface without cross effect in the condition of $\beta_{1}=\beta_{2}=0$. θ is the angle between the preloading direction and the reloading direction, and it is defined by the deviatoric stress and back stress:

(19) $$\theta =\arccos\left(\frac{s-\alpha }{\left\|s-\alpha \right\|}\cdot \frac{\alpha }{\left\|\alpha \right\|}\right).$$

Defining the two flow tensors as:

(20) $$n_{1}=\frac{s-\alpha }{\left\|s-\alpha \right\|},\;n_{2}=\frac{\alpha }{\left\|\alpha \right\|}.$$

Substituting Equation (20) into (19), Equation (19) can be simplified as:

(21) $$\theta =\arccos\left(n_{1}\cdot n_{2}\right).$$

Following the orthogonal flow rule, the plastic flow direction of anisotropic yield surface can be described by the partial derivative of Equation (15):

(22) $$n=\frac{\partial F_{y}}{\partial \sigma }=\frac{s-\alpha }{\left\|s-\alpha \right\|}-\left(\sigma_{0}+R\right)\frac{\partial H}{\partial \sigma }.$$

Because H is 1 and $\frac{\partial H}{\partial \sigma }=0$ under preloading and unloading [given in Equation (16)], the plastic flow direction is orthogonal to the cylindrical yield surface during the two stages, i.e., $n=n_{1}$. Similarly, $\frac{\partial H}{\partial \sigma }=0$ and $n=n_{1}$ are established when anisotropy disappears in the process of reloading, i.e., $\left\|\alpha -\alpha_{unloading}\right\|\geq w\cdot \left\|\alpha_{unloading}\right\|$. The yield surface evolves from anisotropy to quasi-isotropy gradually in the condition of $\left\|\alpha -\alpha_{unloading}\right\|\geq w\cdot \left\|\alpha_{unloading}\right\|$ under reloading, as displayed in Figure 3. Combining Equations (16), (20), and (22), the plastic flow direction n can be expressed as:

(23) $$n=n_{1}-\left(\sigma_{0}+R\right)\left(1-\frac{\left\|\alpha -\alpha_{unloading}\right\|}{w\cdot \left\|\alpha_{unloading}\right\|}\right)\frac{\partial d}{\partial \sigma }.$$

Based on the definition of anisotropic distortional factor d in Equation (18), $\frac{\partial d}{\partial \sigma }$ can be obtained by:

(24) $$\frac{\partial d}{\partial \sigma }=\frac{\partial d}{\partial \left(s-a\right)}=\left(3\beta_{1}\left(1+\beta_{2}\right)\cos^{2}\theta -2\beta_{2}\cos\theta -5\beta_{1}\beta_{2}\cos^{4}\theta \right)\frac{\partial \left(\cos\theta \right)}{\partial \left(s-a\right)}.$$

Due to the definition of $n_{2}$ in Equation (20), which is irrelevant to s−a, the expression can be deduced as:

(25) $$\frac{\partial n_{2}}{\partial \left(s-a\right)}=0.$$

Therefore,

(26) $$\frac{\partial \left(\cos\theta \right)}{\partial \left(s-a\right)}=\frac{\partial \left(n_{1}:n_{2}\right)}{\partial \left(s-a\right)}=\frac{\partial n_{1}}{\partial \left(s-a\right)}:n_{2}.$$

Integrating Equations (23), (24), and (26), the plastic flow direction n can be summarized as:

(27) $$n=\left\{ \begin{array}{cc} n_{1}, & Preloading\;and\;unloading\\ n_{1}-\left(\sigma_{0}+R\right)\left(3\beta_{1}\left(1+\beta_{2}\right)\cos^{2}\theta -2\beta_{2}\cos\theta -5\beta_{1}\beta_{2}\cos^{4}\theta \right) & \\ \cdot \left(1-\frac{\left\|\alpha -\alpha_{unloading}\right\|}{w\cdot \left\|\alpha_{unloading}\right\|}\right)J:n_{2}, & Reloading\;and\;\frac{\left\|\alpha -\alpha_{unloading}\right\|}{w\cdot \left\|\alpha_{unloading}\right\|}\leq 1\\ n_{1} & Reloading\;and\;\frac{\left\|\alpha -\alpha_{unloading}\right\|}{w\cdot \left\|\alpha_{unloading}\right\|}\geq 1 \end{array}\right.,$$

where the stress tensors $n_{1}$ and $n_{2}$ are normalized according to back stress tensor α and stress tensor s−α in Equation (20). J is a fourth-order tensor which can be defined as:

(28) $$J=\frac{Ι-n_{1}⊗n_{1}}{\left\|s-\alpha \right\|}.$$

## 3. Simulation Procedure, Model Calibration, and Data Processing

### 3.1. Specimen Geometry and Finite Element Model

**Figure 4:**
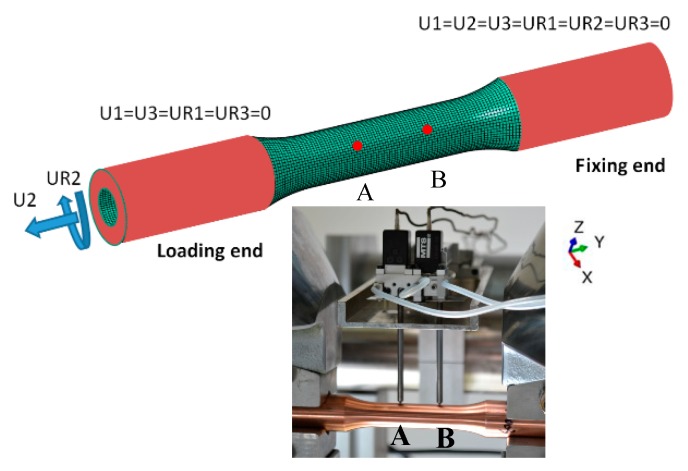
The FEM (Finite Element Method) model of thin cylinder specimen and the experimental tension-torsion strains acquisition method.

The deformation of a thin-walled circular tube specimen, which had a gauge length of 50 mm, a wall thickness of 1 mm, and an outer diameter of 16.5 mm, was simulated. The full-scale geometric model was established and divided into 64640 elements with 77952 nodes, as shown in Figure 4. Five elements were discretized along the direction of wall thickness during the gauge segment. The element type was C3D8R three-dimensional eight-node reduced integration element, which could avoid shear locking and improved the computational efficiency. The model was constrained and loaded by coupling the chuck elements (cf. the red region in Figure 4) with the displacement of the reference point, as shown in Figure 4. The first, third, fourth, and sixth degrees of freedom in the loading end were set to zero, and the axial and rotational displacements were performed on the second and fifth degrees of freedom to realize the combination loading of tension and torsion. All degrees of freedom at the fixed end were set to zero. Based on the finite element platform ABAQUS, the subroutine of user defined material (Umat) was applied to simulate the coupling behavior under tension-torsion loading.

### 3.2. Loading Path for Yield Stress Test

The subsequent yield surfaces of metals were tested by the single-specimen method and the multiple-specimen method. The yield stress was determined by the offset-strain method, which required the elastic modulus calculated from the linear segment of stress-strain curve. Then, the stress corresponding to the specified plastic strain (offset strain) was obtained. The test and the simulation of polycrystalline plastic model showed that the yield point test of single-specimen method was affected by the accumulated plastic deformation under different loading paths and was closely related to the reloading sequence. The dispersion of test data from multiple-specimen method could be controlled by sufficient specimens even though the properties of the material were scattered between specimens. Therefore, the test results based on the multiple-specimen method were used to compare with the simulation results of the anisotropic model in this paper. With the multiple-specimen method, the single specimen was used to determine the different yield stresses on the three offset strains along one reloading direction.

In order to investigate the applicability of anisotropic distortional yield surface model under different preloading paths, the subsequent yield surfaces of thin-walled tube specimens of pure copper under three typical pre-deformation conditions (pre-tension ε=1%, pre-torsion γ3=1%, and pre-tension-torsion proportional loading $\varepsilon =\frac{\gamma }{\sqrt{3}}=\frac{\sqrt{2}}{200}$) were predicted.

**Figure 5:**
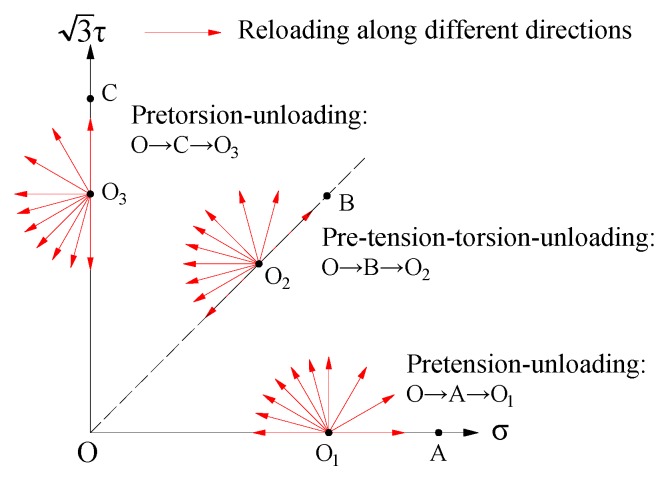
Schematic of probing path for subsequent yield surface under pretension, pretorsion, and pre-tension -torsion loading.

As the unloading process of metals after plastic deformation was nonlinear, and the nonlinearity induced by plastic pre-deformation under loading and unloading process was more prominent, the test data of elastic stage in unloading section was selected. The equivalent stress of the unloading point was 61 MPa (nominal stress) corresponding to the equivalent strain of 1% under preloading. The displacement boundary and force boundary were used to realize the strain control and stress control, respectively. The specimens were firstly subjected to the equivalent strain of 1% under preloading and unloaded to the equivalent stress of 61 MPa and then reloaded along 0, 30, 60, 90, 105, 120, 135, 150, 165, and 180 angles, as depicted in Figure 5. By adopting the continuation method [16], the measured yield stresses along 0, 30, 60, 90, 105, 120, 135, 150, 165, and 180 angles were symmetrically extended to the opposite side of the pre-deformation line. Consequently, the yield points whose reloading paths were along −30, −60, −90, −105, −120, −135, −150, and −165 angles could be obtained easily.

### 3.3. Data Processing Method

The stress during the gauge segment of a thin wall circular pipe was distributed uniformly under the tension-torsion loading. The displacements of the two nodes set in the middle of the gauge segment were simulated with the output of the extensometer, as shown in Figure 4. The Python script file of ABAQUS was edited to deal with and output the variables. The axial displacement, torsional displacement, axial force, and torque of the two reference points in each increment were recorded, and the total strain, plastic strain, direct stress, shear stress, equivalent strain, and equivalent stress were calculated and output by the state variables from the program. Due to the limitation of increments in ABAQUS standard simulation, the yield stresses under different offset strains were automatically obtained using a linear interpolation program by a few results output on different increments. The axial stress, shear stress, and torsional strain in simulation can be calculated as follows:

(29) $$\sigma =\frac{4F}{\pi (D^{2}-d^{2})},$$

(30) $$\tau =\frac{16TD}{\pi (D^{4}-d^{4})},$$

(31) $$\varepsilon =\frac{U_{2}^{A}-U_{2}^{B}}{AB},$$

(32) $$\gamma =\frac{\bar{R}\left(UR_{2}^{A}-UR_{2}^{B}\right)}{AB},$$

where F and T denote axial force and torque, respectively, D is the outer diameter, and d is the internal diameter of the thin-walled tube specimens of the gauge section. $U_{2}^{A},\;U_{2}^{B}$ are the axial displacements of points A and B, and $UR_{2}^{A},\;UR_{2}^{B}$ are the torsional displacements of points A and B, as shown in Figure 4. $\bar{R}$ is the mean radius in gauge segment, and AB is corresponding to the gauge length of extensometer.

Based on Equations (29)–(32), the equivalent stress and strain under the von Mises theory can be defined as:

(33) $$\varepsilon_{eq}=\sqrt{\varepsilon^{2}+\frac{\gamma^{2}}{3}}.$$

Using Hooke’s law, plastic strains can be approximately expressed as:

(34) $$\varepsilon^{p}=\varepsilon -\frac{\sigma }{E},$$

(35) $$\gamma^{p}=\gamma -\frac{\tau }{G},$$

(36) $$\varepsilon_{eq}^{p}=\sqrt{{\left(\varepsilon^{p}\right)}^{2}+\frac{{\left(\gamma^{p}\right)}^{2}}{3}},$$

where E and G are elastic modulus and shear modulus, respectively.

### 3.4. Calibration of Model

#### 3.4.1. Calibration of Anisotropic Parameters

**Figure 6:**
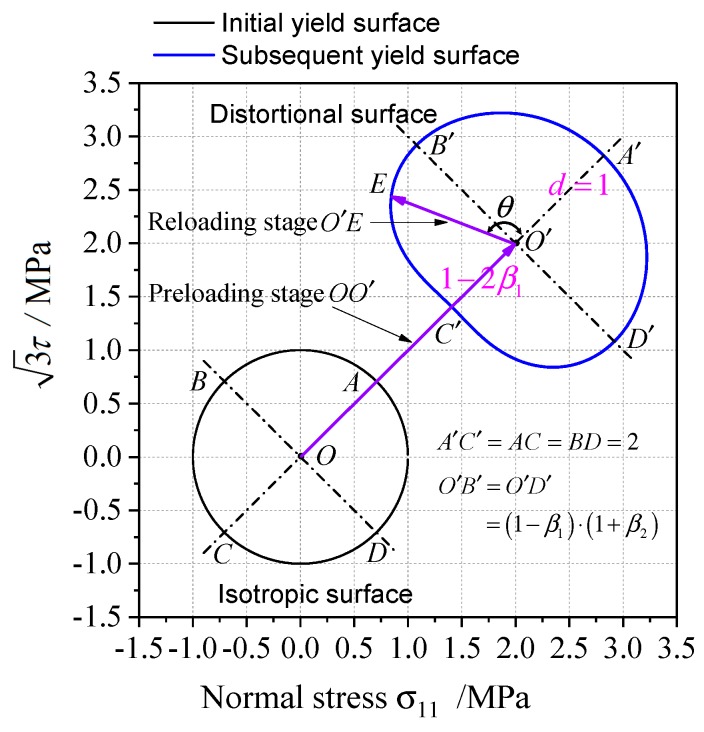
Schematic of anisotropic distortion subsequent yield surface on $\sigma -\sqrt{3}\tau$ stress plane.

It can be analyzed from Equations (18) and (21) that the anisotropic distortional factor d changes with $n_{1}\cdot n_{2}$. The anisotropic hardening model in this paper is calibrated by the tension-torsion tests. Assume the stress state of unloading after preloading is $\sigma^{*}$ and the reloading path is $\sigma -\sigma^{*}$, $\sigma^{*}$ can be simplified to $O^{\prime }$ under tension-torsion stress state, which is the center of the subsequent yield line (cf. the blue solid line in Figure 6), and the preloading-unloading path is $O\to A^{\prime }\to O^{\prime }$. The reloading path $\sigma -\sigma^{*}$ corresponds to the vector $\vec{O^{\prime }E}$ on two-dimensional stress plane. The angle between preloading path and reloading path can be denoted as $∠A^{\prime }O^{\prime }E$, as shown in Figure 6. Observe that the angle θ between preloading path and reloading path equals the angle between $n_{1},\;n_{2}$ defined by Equation (21) in $s_{11}-\sqrt{3}s_{12}$ deviatoric stress space. Therefore, the cross effect parameters $\beta_{1},\beta_{2}$ can be calibrated by the characteristic of distortional subsequent yield surface under tension-torsion loading.

If the distortional yield surface exhibits prominent cross effect (the sharp point in pre-deformation direction and the relatively flat bottom at its opposite direction), the yield surface can be roughly symmetric with the proportional preloading direction in $\sigma -\sqrt{3}\tau$ stress space. As the subsequent yield surface $\overset{⌢}{A^{\prime }B^{\prime }C^{\prime }D^{\prime }}$ is symmetric with respect to $A^{\prime }C^{\prime }$ in Figure 6, $O^{\prime }B^{\prime }=O^{\prime }D^{\prime }=\left(1-\beta_{1}\right)\left(1+\beta_{2}\right)$. $O^{\prime }$, which is the center of the distortional yield surface, is the intersection of $B^{\prime }D^{\prime }$ and $A^{\prime }C^{\prime }$, therefore $O^{\prime }A^{\prime }=1$, $O^{\prime }C^{\prime }=1-2\beta_{1}$. $B^{\prime }$ and $D^{\prime }$ are the points of maximum distance from the symmetry axis. The anisotropic model parameters $\beta_{1},\beta_{2}$ can be calibrated by the measured yield surface as:

(37) $$\beta_{1}=\frac{O^{\prime }A^{\prime }-O^{\prime }C^{\prime }}{2\cdot O^{\prime }A^{\prime }},\;\beta_{2}=\frac{B^{\prime }D^{\prime }}{A^{\prime }C^{\prime }}-1\;.$$

The cross effect parameters $\beta_{1},\beta_{2}$ of distortional yield surface can describe the characteristics of anisotropic yield. The directional distortion and cross effect of subsequent yield surface are more distinct as $\beta_{1}$ increases. In particular, the yield surface expands in the direction perpendicular to the preloading path as $\beta_{2}>1$, the yield surface shrinks in the direction perpendicular to the preloading path as $\beta_{2}<1$, and the inner diameter of the yield surface keeps constant in the direction perpendicular to the preloading path as $\beta_{2}=1$.

#### 3.4.2. A Representative Volume Element for the Calibration of Parameters in Anisotropic Constitutive Model

**Figure 7:**
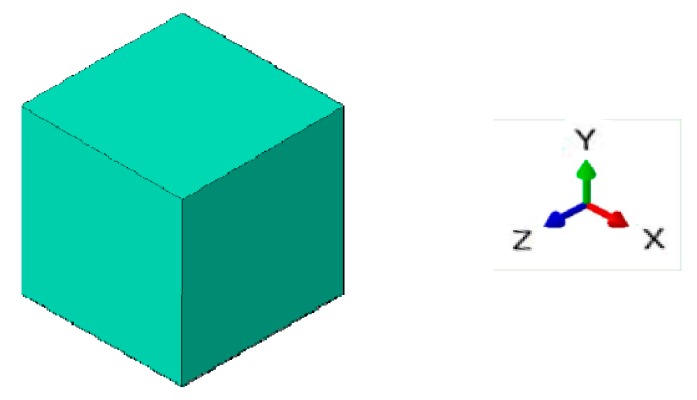
Representative volume element for the simulation with unique element.

The above anisotropic constitutive model proposed was implemented as a user defined material subroutine (UMAT) into the ABAQUS/Standard module. In order to rapidly determine the model parameters, a representative volume element (1 mm × 1 mm × 1 mm) was adopted, as shown in Figure 7. The external surface of the representative element kept plane after deformation due to the specific boundary condition. By fitting the computationally obtained cyclic stress-strain curve to the experimentally measured one, the material parameters of anisotropic plastic model for T2 copper under monotonic loading and cyclic loading are estimated as Table 1.

**Figure 8:**
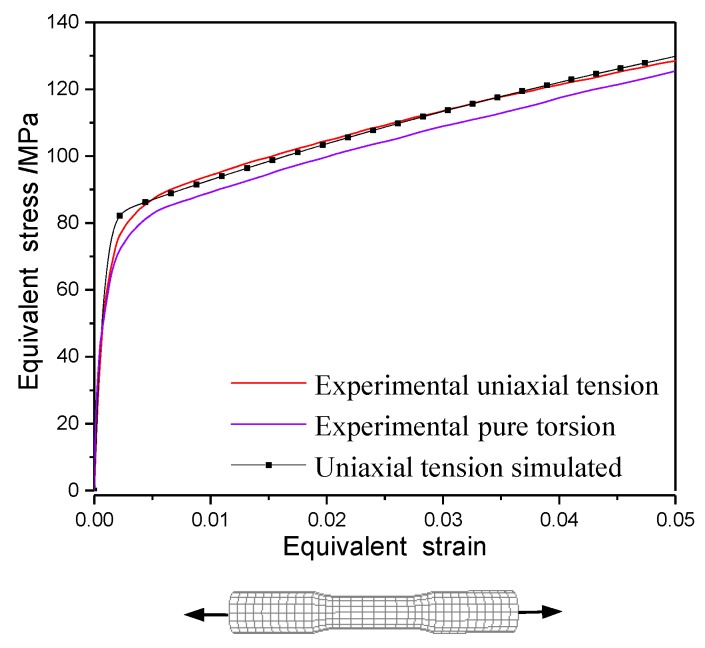
The stress-strain curves of T2 pure copper under uniaxial tension and pure torsion by test [33] and simulation with the anisotropic model.

**Figure 9:**
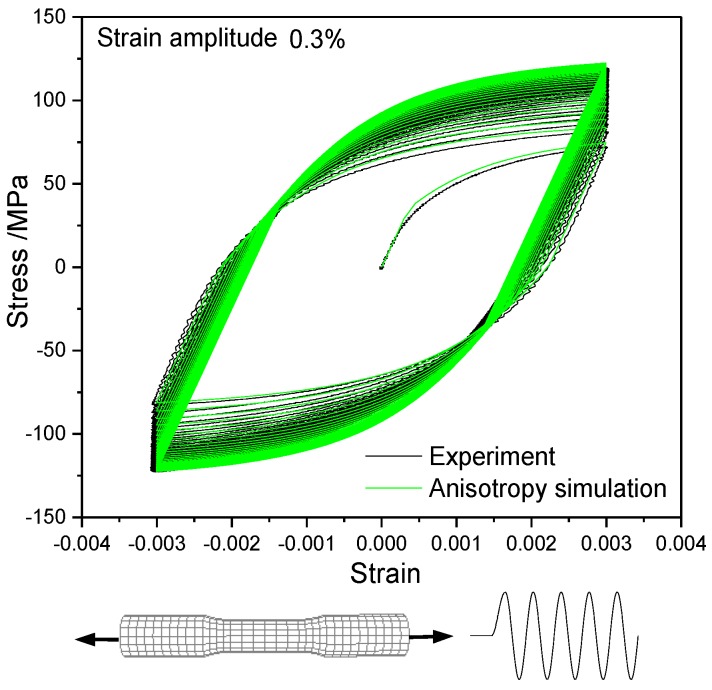
Simulation for the experimental hysteresis loop [33] of uniaxial tension- compression with the anisotropic model.

To validate the precision of the anisotropic plastic model, the simulated stress-strain curve of T2 pure copper under uniaxial tension and the hysteresis loop under uniaxial tension-compress with symmetric strain amplitudes were compared with the experimentally measured ones in Figure 8 and Figure 9, respectively. The results indicate that the macroscopic mechanical behaviors of the materials under monotonic loading and cyclic loading simulated by the anisotropic constitutive model were in good agreement with the experimental results. The equivalent stress-strain curves obtained by the uniaxial tensile test and the torsion test with thin-walled circular pipe specimen were different, as indicated in Figure 8. All model parameters in this paper were calibrated by the pre-tension test. The equivalent stress-strain curve simulated by the anisotropic plastic model was fully consistent with the experimental one under tension, but the equivalent stress-strain curve simulated by the anisotropic plastic model was slightly higher than the experimental data under torsion of thin-walled circular pipe specimen.

## 4. Simulation of Anisotropic Subsequent Yield Surface

**Figure 10:**
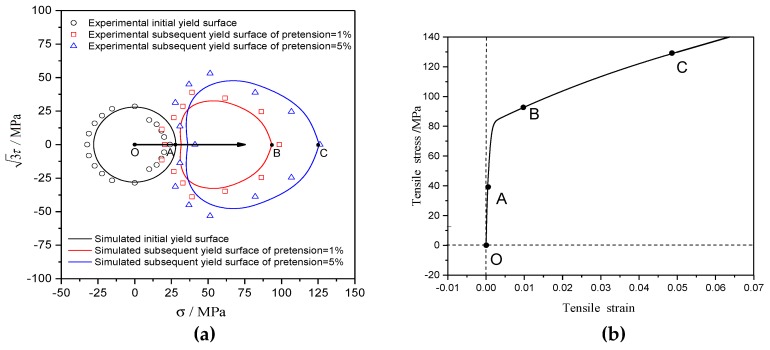
(**a**) The experimental yield surfaces with different pretension strains of 1% and 5% [34] versus the results simulated; (**b**) the stress-strain relationship corresponds to the path O-A-B-C.

The subsequent yield surface of T2 pure copper under pretension loading exhibited significant anisotropy [34], as shown in Figure 10. According to the test method described in Section 3, the initial yield surface with the offset strain of 5 × 10^−5^ was nearly circular when the material yielded firstly under pretension loading [cf. the point A in Figure 10a,b]. T2 pure copper began to harden when the equivalent pre-tensile strains increased to 1% and 5% [cf. the point B and point C in Figure 10a,b], and the subsequent yield surface [cf. the blue triangle and red rectangular in Figure 10a] was larger than the initial yield surface [cf. the black circle in Figure 10a]. The subsequent yield surface was characterized by a region of high curvature along the direction of preloading and a region of flattening on the opposite side. The yield surface calculated by the anisotropic model in this paper [cf. the blue, red, and black curves in Figure 10a] were in good agreement with the experimental results, which indicates that this model can describe the anisotropic distortional characteristics of the subsequent yield surface.

The simulation results of the anisotropic distortional yield surface model were compared with the experimental data and the simulation results of polycrystalline plastic model [16] to discuss the subsequent yield and hardening process of metal materials. In particular, the yield stresses determined by the very small offset strain showed significant dispersion, which gradually decreased as the offset strain increased. Thus the typical offset strains of 1 × 10^−4^, 5 × 10^−4^, and 1 × 10^−3^ were adopted to investigate the subsequent yield behavior.

**Figure 11:**
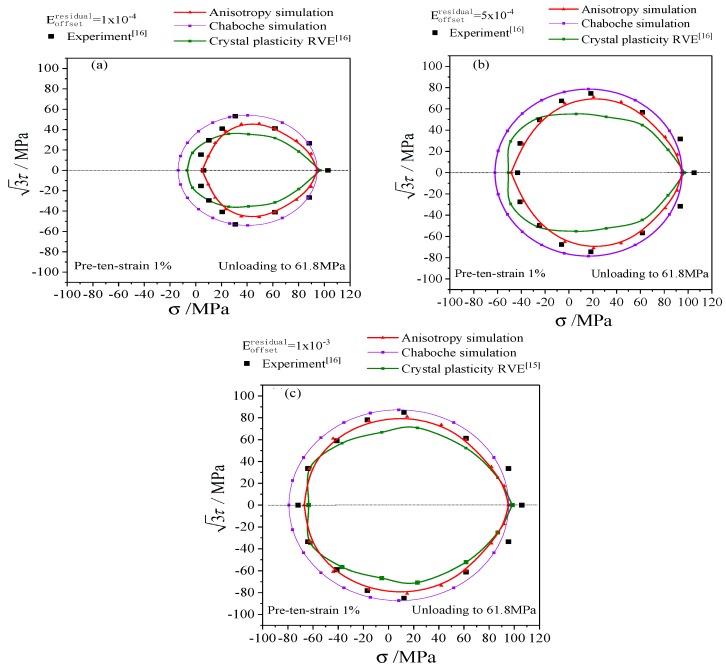
The simulations and experiments of subsequent yield surfaces on the $\sigma -\sqrt{3}\tau$ stress plane with preloading of 1% proportional pretension equivalent strain and unloading to equivalent stress of 61.8 MPa: (**a**) the offset strain is 1 × 10^−4^; (**b**) the offset strain is 5 × 10^−4^; (**c**) the offset strain is 1 × 10^−3^.

**Figure 12:**
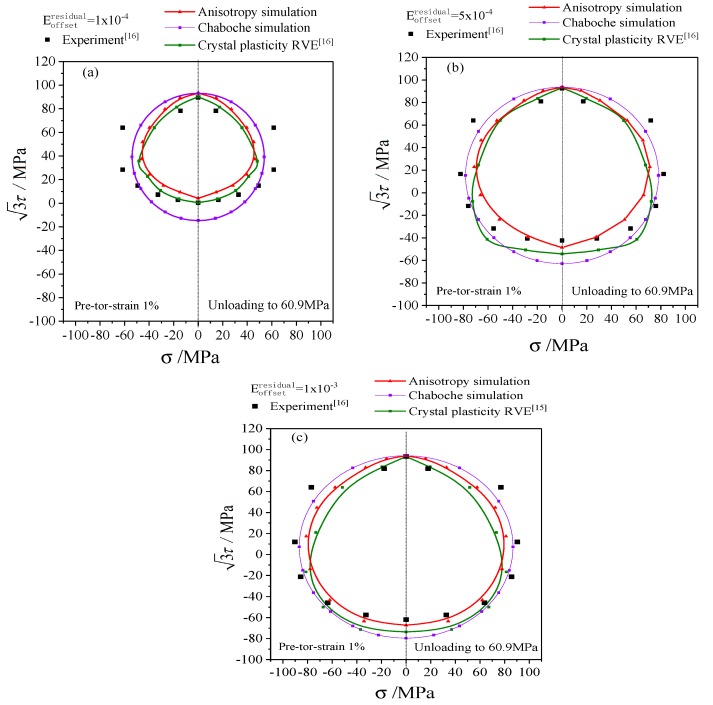
The simulations and experiments of subsequent yield surfaces on the $\sigma -\sqrt{3}\tau$ stress plane with preloading of 1% proportional pretorsion equivalent strain and unloading to equivalent stress of 60.9 MPa: (**a**) the offset strain is 1 × 10^−4^; (**b**) the offset strain is 5 × 10^−4^; (**c**) the offset strain is 1 × 10^−3^.

**Figure 13:**
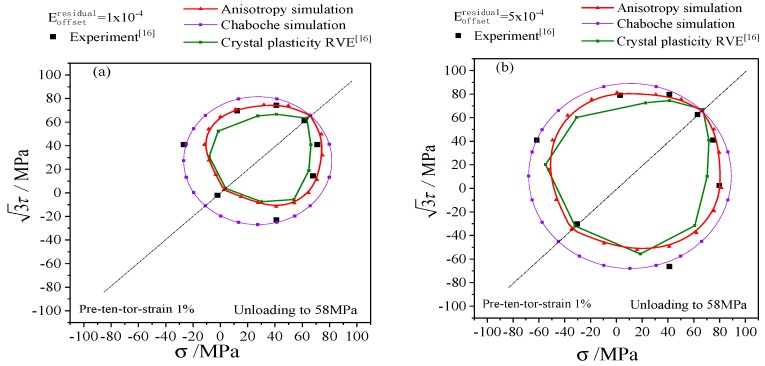

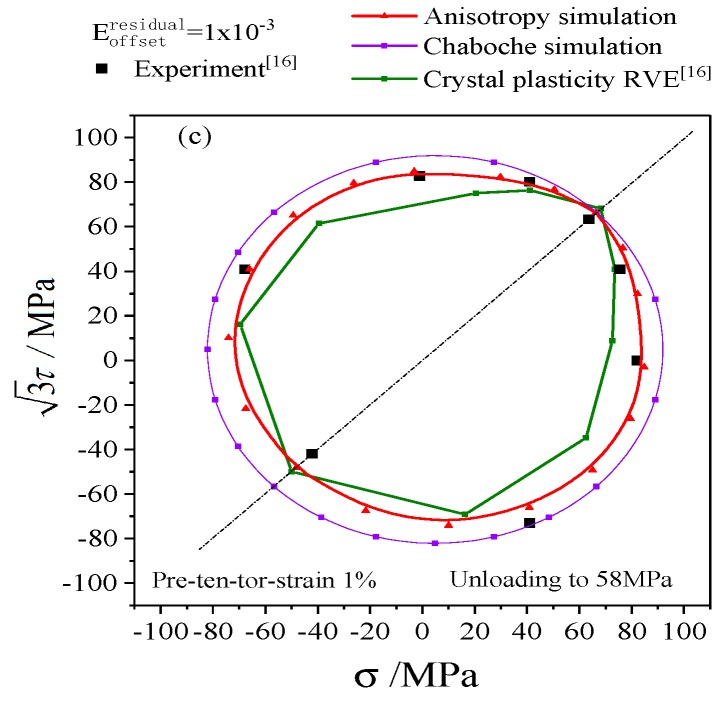
The simulations and experiments of subsequent yield surfaces on the $\sigma -\sqrt{3}\tau$ stress plane with preloading of 1% proportional pre-tension-torsion equivalent strain and unloading to equivalent stress of 58 MPa: (**a**) the offset strain is 1 × 10^−4^; (**b**) the offset strain is 5 × 10^−4^; (**c**) the offset strain is 1 × 10^−3^.

The subsequent yield surfaces determined by unloading and proportional reloading after three directions of pre-deformations (tensile, torsion, and tension-torsion) are shown in Figure 11, Figure 12 and Figure 13 respectively, which includes yield stresses corresponding to the typical offset strains of 1 × 10^−4^ [Figure 11a, Figure 12a and Figure 13a], 5 × 10^−4^ [Figure 11b, Figure 12b and Figure 13b], 1 × 10^−3^ [Figure 11c, Figure 12c and Figure 13c]. Since the classical Chaboche plastic constitutive model only includes kinematic hardening and isotropic hardening, the subsequent yield surface calculated by Chaboche model without distortional hardening is a circle with the center of back stress, as shown in Figure 11, Figure 12 and Figure 13.

The anisotropic distortional yield surface expanded continuously with the increase of offset strain, which was consistent with the experimental data and the simulation results of the crystal plastic model. The cross effect of the anisotropic yield surface was relatively prominent with the offset strain of 1 × 10^−4^. The “sharp point” appeared in the preloading direction, and the yield surface was flat in the opposite direction. The cross effect of the distortional yield surface was gradually weakened when the offset strain increased from 1 × 10^−4^ to 5 × 10^−4^ and eventually reached 1 × 10^−3^, and the anisotropic distortional yield surface was gradually approaching the isotropic circular yield surface computed by the Chaboche model (cf. the purple solid lines in Figure 11, Figure 12 and Figure 13), which complied with the experimental data.

Comparing the results of the crystal plastic constitutive model (cf. the green lines in Figure 11, Figure 12 and Figure 13) with the results of anisotropic distortional yield surface model shows that both of them could describe the expansion, shift, and distortion of subsequent yield surface, but the results of the anisotropic model in this paper were generally better than those of the crystal plastic constitutive model. The simulation results of the anisotropic model were very close to the experimental data under pre-tension, which was superior to the simulation results of crystal plasticity model, as presented in Figure 11. The results of the anisotropic model were slightly higher than the experimental values in the case of pre-torsion and pre-tension-torsion, and the crystal plastic model was more accurate along the torsional direction. However, the yield stress perpendicular to the preloading direction for the crystal plastic model in Figure 12 and Figure 13 was not as precise as that of the anisotropic model.

The parameters of kinematic hardening and isotropic hardening parts of anisotropic yield model in Table 1 are the same as those in Chaboche model. The diameter of the anisotropic distortional yield surface (cf. the red solid lines in Figure 11, Figure 12 and Figure 13) was smaller than that of the circular yield surface simulated by Chaboche model under small offset strains, and the ratio of the two diameters along the loading direction was $R_{distortion}:R_{circle}=1:\left(1+\beta_{1}\right)$, as shown in Figure 11, Figure 12 and Figure 13. Therefore, the subsequent yield surface derived from the Chaboche model was the same as the subsequent yield surface simulated by the anisotropic yield model when anisotropy gradually degenerated to isotropy. The diameter of the circular yield surface simulated by Chaboche model could be reduced to fit the experimental curve by adjusting the parameters of the model, and the simulation results were obviously better than the curves with the parameters adopted in this paper. However, the non-circular characteristic of the experimental yield surface could not be well captured by Chaboche model.

Due to the different numbers of the experimental data of the three pre-loading conditions, the error of each yield surface determined by different offset strain under various pre-deformation conditions is defined based on the benchmark of the experimental yield stresses as:

(38) $$err_{aniso}{}^{ij}=\frac{1}{N}\sum_{k=1}^{N}\left|\frac{{\left\|\sigma_{aniso}-\sigma_{test}\right\|}_{2}{}^{k}}{{\left\|\sigma_{test}-\sigma_{unloading}\right\|}_{2}{}^{k}}\right|,\;err_{cryst}{}^{ij}=\frac{1}{N}\sum_{k=1}^{N}\left|\frac{{\left\|\sigma_{cryst}-\sigma_{test}\right\|}_{2}{}^{k}}{{\left\|\sigma_{test}-\sigma_{unloading}\right\|}_{2}{}^{k}}\right|,$$

where the $err_{aniso}{}^{ij}$ and $err_{aniso}{}^{ij}$ represent, respectively, the mean errors of the anisotropic model and the crystal plastic model with the i-th offset strain and the j-th pre-deformation type, the N is the total number of reloading directions, the k indicates the k-th reloading direction, and ${\left\|\right\|}_{2}$ expresses the 2-norm (Euclid norm) of the stress tensor.

The mean value of every error for the anisotropic model and the crystal plastic model are given respectively by:

(39) $$\bar{err_{aniso}}=\frac{1}{9}\sum_{i,j=1}^{3}err_{aniso}{}^{ij},\;\bar{err_{cryst}}=\frac{1}{9}\sum_{i,j=1}^{3}err_{cryst}{}^{ij},$$

where the $\bar{err_{aniso}}$ and $\bar{err_{cryst}}$ are the mean error of the anisotropic model and the crystal plastic model, and the total numbers of i and j are both three.

The standard deviation is calculated with fundamental statistics knowledge by the error and the mean error. The errors and the standard deviation of the yield surface with the anisotropic model and the crystal plastic model under every offset strain and pre-formation are listed in Table 2. There was an obvious difference in the predicted accuracy between various offset strains and pre-formations conditions. In terms of the pre-torsion loading, the mean error and the standard deviation of the anisotropic model were higher than those of the crystal plastic model, which was attributed to the shear hardening model in slip process. On the whole, however, the mean error and the standard deviation of the anisotropic model, 11.54% and 8.23%, were both smaller than those of the crystal plastic model, as shown in the last column of Table 2.

## 5. Discussion and Conclusions

Compared with the experimental data and the simulated results of crystal plastic model, the anisotropic distortional yield surface model proposed in this paper can better exhibit the anisotropic yield characteristic after preloading and the cross effect of subsequent yield surface. The anisotropic distortional yield surface model can characterize the nonlinear hardening process that anisotropic yield surface expands to isotropic yield surface under reloading. The predicted accuracy of the anisotropic model is higher than the crystal plastic model, with the mean error and the standard deviation as 11.54% and 8.23%, which proves the reasonability and validity of the anisotropic distortional yield surface model to capture the anisotropic distortional features.

Considering the cross effect, the anisotropic yield model can accurately simulate the experimental yield stresses, which are used to calibrate the parameters of the anisotropic model (here, it is calibrated by the experimental results under pre-tension loading). Nevertheless, there was a certain deviation between the yield surface predicted and the experimental data under other preloading conditions. The anisotropic yield model was developed based on the Chaboche model within the framework of the von Mises theory. Meanwhile, the equivalent stress-strain curve under pure torsion loading was lower than that of uniaxial tension (shown in Figure 8). Therefore, the yield stresses predicted by the anisotropic yield model were higher than those of the experimental ones under pre-torsion and pre-tension-torsion loading. The cross effect of subsequent yield surface varies with preloading conditions according to the experimental data and simulation results are shown in Figure 11, Figure 12 and Figure 13.

It should be pointed out that some combinations of the two cross effect parameters $\beta_{1},\beta_{2}$ in anisotropic yield model can describe the concave yield surface, which is consistent with the concavity of experimental yield surface under pre-deformation strain of 5% for T2 pure copper [34]. However, it is contrary to the convexity hypothesis of the loading surface deduced by the fundamental Drucker postulate within the classical plastic theory framework, which implies that further study in relation to the concavity of yield surface needs to be performed.

## Figures and Tables

**Table 1:** Material parameters of anisotropic plastic model for T2 copper.

	Elastic Constants		Isotropic Hardening Parameters					Kinematic Hardening Parameters			Anisotropic Hardening Parameters			
	Ε	ν	b	Q	$\sigma_{0}$	K	m	$a_{1}$	$C_{1}$	$a_{2}$	$C_{2}$	$\beta_{1}$	$\beta_{2}$	w
unit	GPa			MPa	MPa	MPa		MPa		MPa				
Anisotropic model	97	0.326	15	50	28	8	100	31	3000	51	7	0.3	0.1	4
Chaboche model	97	0.326	15	50	28	8	100	31	3000	51	7	0	0	0

**Table 2:** The errors and the standard deviation of the yield surface with the anisotropic model and the crystal plastic model.

Error (%)	Pre-Tension Loading			Pre-Torsion Loading			Pre-Ten-tor Loading			Mean (Standard Deviation)
	1 × 10^−4^	5 × 10^−4^	1 × 10^−3^	1 × 10^−4^	5 × 10^−4^	1 × 10^−3^	1 × 10^−4^	5 × 10^−4^	1 × 10^−3^	
Crystal plasticity RVE	18.64 (11.09)	17.1 (10.73)	12.67 (7.99)	14.0 (14.33)	12.61 (8.61)	11.5 (10.19)	15.5 (5.41)	15.16 (8.8)	15.29 (7.28)	14.72 (9.38)
Anisotropy simulation	9.91 (6.98)	8.24 (9.42)	8.08 (10.07)	20.83 (12.93)	13.05 (10.95)	11.28 (11.2)	14.1 (4.66)	9.74 (5.32)	8.65 (2.56)	11.54 (8.23)

## References

[1] Ohashi Y., Kawashima K., Yokochi T. (1975). Anisotropy due to plastic deformation of initially isotropic mild steel and its analytical formulation. J. Mech. Phys. Solids.

[2] Cheng S., Krempl E. (1991). Experimental determination of strain-induced anisotropy during nonproportional straining of an A1/Mg alloy at room temperature. Int. J. Plast..

[3] Prager W. (1949). Recent developments in the mathematical theory of plasticity. J. Appl. Phys..

[4] Ziegler H. (1959). A modification of prager’s hardening rule. Q. Appl. Math..

[5] Mróz Z. (1967). On the description of anisotropic workhardening. J. Mech. Phys. Solids.

[6] Hecker S.S. (1976). Constitutive equations in viscoplasticity:Computational and engineering aspects. Am. Soc. Mech. Eng..

[7] Ohno N., Wang J.D. (1991). Transformation of a nonlinear kinematic hardening rule to a multisurface form under isothermal and nonisothermal conditions. Int. J. Plast..

[8] Chaboche J.L., Dang Van K., Cordier G. Modelization of the strain memory effect on the cyclic hardening of 316 stainless steel. Proceedings of the Transactions of the 5th International Conference on Structural Mechanics in Reactor Technology.

[9] Chaboche J.L. (1991). On some modifications of kinematic hardening to improve the description of ratchetting effects. Int. J. Plast..

[10] Chaboche J.L. (1994). Modeling of ratchetting: Evaluation of various approaches. Eur. J. Mech. A-Solids.

[11] Armstrong P., Frederick C.O. (2007). A mathematical representation of the multiaxial Bauschinger effect. High Temp. Technol..

[12] Phillips A., Tang J.L. (1972). The effect of loading path on the yield surface at elevated temperatures. Int. J. Solids Struct..

[13] Wu H.C., Yeh W.C. (1991). On the experimental determination of yield surfaces and some results of annealed 304 stainless steel. Int. J. Plast..

[14] Khan A.S., Wang X.W. (1993). An experimental study on subsequent yield surface after finite shear prestraining. Int. J. Plast..

[15] Hu G.J., Huang S.H., Lu D.M., Zhong X.C., Li Z.H., Brocks W., Zhang K.S. (2015). Subsequent yielding of polycrystalline aluminum after cyclic tension–compression analyzed by experiments and simulations. Int. J. Solids Struct..

[16] Zhang K.S., Huang S.H., Liu G.J., Lu D.M. (2017). Measuring subsequent yield surface of pure copper by crystal plasticity simulation. Chin. J. Theor. Appl. Mech..

[17] Hu G.J. (2012). Plastic Behavior of Metals under Tension-Torsion Loading-Exprerimental and Numerical Research on Yield Surface Evolution. Ph.D. Thesis.

[18] Khan A.S., Pandey A., Stoughton T. (2010). Evolution of subsequent yield surfaces and elastic constants with finite plastic deformation. Part II: A very high work hardening aluminum alloy (annealed 1100 Al). Int. J. Plast..

[19] Vincent L., Calloch S., Marquis D. (2004). A general cyclic plasticity model taking into account yield surface distortion for multiaxial ratchetting. Int. J. Plast..

[20] Feigenbaum H.P., Dafalias Y.F. (2007). Directional distortional hardening in metal plasticity within thermodynamics. Int. J. Solids Struct..

[21] Feigenbaum H.P., Dugdale J., Dafalias Y.F., Kourousis K.I., Plesek J. (2012). Multiaxial ratcheting with advanced kinematic and directional distortional hardening rules. Int. J. Solids Struct..

[22] Shi Y.K., Zhang K.S., Hu G.J. (2009). Subsequent yield and plastic flow analysis of polycrystalline copper under biaxial loading. Acta Metall. Sin..

[23] Tadano Y., Yoshihara Y., Hagihara S. (2016). A crystal plasticity modeling considering volume fraction of deformation twinning. Int. J. Plast..

[24] Francois M. (2010). A plasticity model with yield surface distortion for non proportional loading. Int. J. Plast..

[25] Shi B.D., Yan P., Chong Y., Pan F.S., Cheng R.J., Peng Q.M. (2017). Loading path dependent distortional hardening of Mg alloys: Experimental investigation and constitutive modeling. Int. J. Plast..

[26] Fu Q., Liu F., Zhang J. (2010). A physically motivated model for the evolution of subsequent yield surfaces. J. Theor. Appl. Mech..

[27] Chen Y., Hu G.J., Lan Y.T., Zhang K.S., Cai G.W. (2018). Constitutive modeling of slip, twinning and detwinning for mg alloy and inhomogeneous evolution of microstructure. Acta Mech. Solida Sin..

[28] Wen W., Borodachenkova M., Tomé C.N., Vincze G., Rauch E.F., Barlat F., Grácio J.J. (2016). Mechanical behavior of low carbon steel subjected to strain path changes: Experiments and modeling. Acta Mater..

[29] Zhang K.S., Wu M.S., Feng R. (2005). Simulation of microplasticity-induced deformation in uniaxially strained ceramics by 3-D Voronoi polycrystal modeling. Int. J. Plast..

[30] Zhang K.S., Shi Y.K. (2011). Anisotropy of yielding/hardening and microinhomogeneity of deforming/rotating for a polycrystalline metal under cyclic tension-compression. Acta Metall. Sin..

[31] Zhang K.S., Shi Y.K., Ju J.W. (2013). Grain-level statistical plasticity analysis on strain cycle fatigue of a FCC metal. Mech. Mater..

[32] Khan A.S., Kazmi R., Pandey A., Stoughton T. (2009). Evolution of subsequent yield surfaces and elastic constants with finite plastic deformation. Part-I: A very low work hardening aluminum alloy (Al6061-T6511). Int. J. Plast..

[33] Huang S.H. (2018). Test and Crystal Plasticity Numerical Study on the Evolution of Metals Subsequent Yield Surface. Ph.D. Thesis.

[34] Liu G.L., Huang S.H., Shi C.S., Zeng B., Zhang K.S., Zhong X.C. (2018). Experimental Investigations on Subsequent Yield Surface of Pure Copper by Single-Sample and Multi-Sample Methods under Various Pre-Deformation. Materials.

